# Sinapic Acid Protects SH-SY5Y Human Neuroblastoma Cells against 6-Hydroxydopamine-Induced Neurotoxicity

**DOI:** 10.3390/biomedicines9030295

**Published:** 2021-03-13

**Authors:** Tsendsuren Tungalag, Dong Kwon Yang

**Affiliations:** Department of Veterinary Pharmacology and Toxicology, College of Veterinary Medicine, Jeonbuk National University, Iksan, Jeollabuk-do 54596, Korea; mgljuuh@gmail.com

**Keywords:** Parkinson’s disease, sinapic acid, oxidative stress, ER stress, mitochondrial dysfunction, MAPK

## Abstract

Parkinson’s disease (PD) is characterized by progressive dopaminergic neuron loss or dysfunction and is the second most prevalent neurodegenerative disorder after Alzheimer’s disease. However, current therapeutic strategies for PD are limited to treating the outcomes of this disease rather than preventing it. Sinapic acid (SA) is a phenolic compound with potential antioxidant properties, which reportedly acts as a therapeutic agent against many diseases including cancer, as well as cardiac and liver diseases. However, little is known about the effects of SA against neurodegenerative disorders. Therefore, our study sought to evaluate the neuroprotective effects of non-cytotoxic concentrations of SA against 6-hydroxydopamine (6-OHDA)-induced neurotoxicity in SH-SY5Y human neuroblastoma cells, which we used as an in vitro PD model. SA increased cell viability and rescued the cells from 6-OHDA-induced apoptotic cell death. Additionally, oxidative stress responses were significantly blocked by SA, including reactive oxygen species (ROS) overproduction and decreased expression levels of antioxidant proteins. Notably, SA also attenuated mitochondrial dysfunction and endoplasmic reticulum (ER) stress. Moreover, SA dramatically inhibited the activation of mitogen-activated protein kinase (MAPK) proteins. Taken together, our findings highlight the potential PD prevention effects of SA, as well as its underlying mechanisms, making this compound a promising prevention and treatment agent for PD.

## 1. Introduction

Neurodegenerative disorders are characterized by the progressive degeneration of neurons in the central or peripheral nervous system, which causes cognitive decline, memory loss, impaired motor function, and dementia [1]. Moreover, given that these diseases are closely related to age, the incidence of neurodegenerative disorders is now rapidly increasing as the elderly population grows [2]. Nevertheless, the causative factors and pathological mechanisms of these diseases are not yet fully understood.

Alzheimer’s disease, Parkinson’s disease, and Huntington’s disease are the three most common neurodegenerative diseases [3]. Among them, Parkinson’s disease (PD) is the second most prevalent neurodegenerative disorder after Alzheimer’s disease, and worldwide PD incidence and prevalence rates are 10–50 and 100–300 per 100,000 people, respectively [4]. Additionally, PD occurrence has also been shown to increase rapidly with age, particularly in individuals over the age of 65 [5]. Therefore, the number of PD patients is expected to double in 2030 as the elderly population continues to grow [6]. PD is characterized by dopaminergic neuron loss in the substantia nigra pars compacta accompanied by the depletion of dopamine, which leads to impaired motor performance [7]. Therefore, PD patients exhibit serious motor dysfunction including rigidity, static tremor, and resting bradykinesia. Although the exact molecular mechanisms of PD are not well understood, several mechanisms have been proven to be associated with PD, including oxidative stress, mitochondrial dysfunction, and protein misfolding and aggregation, among others [8,9].

Mitochondria are important cellular organelles that produce Adenosine triphosphate (ATP) (i.e., the primary source of energy in cells) through oxidative phosphorylation. This process involves the transfer of electrons from NADH (nicotinamide adenine dinucleotide) or FADH_2_ (flavin adenine dinucleotide) to O_2_, which is mediated by complex I to IV of the electron transport chain. During this process, the interaction between unpaired electrons and molecular O_2_ produces the superoxide radical, which is the primary reactive oxygen species (ROS) in mitochondria [10]. The superoxide radical is further converted into hydrogen peroxide (H_2_O_2_), which is then released from the mitochondria into the cytosol, causing further cellular damage (i.e., oxidative stress) if not eliminated in time [11]. The central nervous system (CNS) contains a uniquely large number of mitochondria to supply its high energy needs [12] and oxidative phosphorylation is the major mechanism of energy production [13]. Therefore, the CNS is particularly susceptible to oxidative stress. Many studies have reported evidence suggesting that oxidative stress plays a crucial role in the pathogenesis of PD [14]. For instance, the CNS exhibits a high iron content, as it is required to maintain the function of mitochondrial enzymes. Furthermore, iron could also lead to ROS overproduction, which contributes to oxidative damage and neuron degeneration, particularly that of nigral dopaminergic neurons [15].

The endoplasmic reticulum (ER) is an intracellular organelle in eukaryotic cells that specializes in protein folding and post-translational maturation of membrane and secreted proteins [16]. However, stressful stimuli (e.g., metabolic disorders, inflammation, disruption of calcium homeostasis, and oxidative stress) lead to the accumulation of unfolded and misfolded proteins in the ER lumen, thus causing ER stress [17]. In turn, the aforementioned process activates the unfolded protein response (UPR) to enhance ER capacity or suppression of misfolded protein synthesis via ER-associated protein degradation (ERAD) and autophagy [18]. However, apoptotic cell death progresses if ER stress is too prolonged or exceeds the capacity of the ERAD response. Several studies have reported that ER stress is involved in the pathogenesis of PD [19]. Treatment with 6-hydroxydopamine (6-OHDA), rotenone, and MPTP (1-methyl-4-phenyl-1,2,3,6-tetrahydropyridine) to mimic PD symptoms activates UPR genes in cell culture systems [20]. However, the ablation of the C/EBP homologous protein (CHOP, i.e., a mediator of ER stress-induced apoptosis) was shown to protect dopaminergic neurons against 6-OHDA treatment in CHOP null mice [21].

Sinapic acid (SA) is a hydroxycinnamic acid-derived polyphenol found in various food crops such as oilseed crops, cereals, and vegetables [22,23]. It possesses pharmacological properties against many diseases such as cancer [24], osteoarthritis [25], hepatic fibrosis [26], and cardiac hypertrophy [27]. More importantly, SA was found to possess neuroprotective effects against Alzheimer’s disease through the inhibition of oxidative stress and inflammation in an amyloid β-induced Alzheimer’s disease mouse model [28]. However, the protective effect of SA against PD has not been characterized.

Therefore, our study sought to elucidate the beneficial effects and possible protective mechanisms of SA against PD in 6-OHDA-treated SH-SY5Y human neuroblastoma cells as a PD cell culture model.

## 2. Materials and Methods

### 2.1. Cell Culture

SH-SY5Y human neuroblastoma cells purchased from the Korean Cell Line Bank (Seoul, Korea) were cultured in Dulbecco’s modified Eagle’s medium (DMEM; GIBCO-BRL, Grand Island, NY, USA) with 10% fetal bovine serum (FBS; GIBCO-BRL), 1% antibiotic cocktail, 2 mM of glutamine. The cells were then grown in a humidified incubator with 5% CO_2_ at 37 °C.

### 2.2. Sinapic Acid and 6-OHDA Treatment in SH-SY5Y Human Neuroblastoma Cells

First, SA (Sigma Chemical Co., St. Louis, MO, USA) was dissolved in 0.1% dimethyl sulfoxide (DMSO; Sigma). The SH-SY5Y cells were then cultured in serum-free medium for 24 h once they reached 80% confluency. Afterward, the cells were treated with 50, 100, 200, 400, and 800 μM of SA for 12, 24, and 48 h to assess SA cytotoxicity. For functional studies, the cells were pretreated with 100, 200, and 400 μM SA for 24 h, followed by 50 μM of 6-hydroxydopamine (6-OHDA; Sigma) treatment for another 12 h to induce neurotoxicity.

### 2.3. Cell Viability Assay

Cell viability was performed via the 3-(4–dimethylthiazol-2-yl)-2,5-diphenyltetrazolium bromide (MTT; Sigma) assay. Briefly, SH-SY5Y cells (1 × 10^4^ cells per well) were seeded in 96-well plates and treated with SA alone or pretreated with SA (100, 200, and 400 μM) for 24 h followed by 50 μM of 6-OHDA for another 12 h. Afterward, the cells were incubated with 500 μg/mL MTT reagent for 2 h at 37 °C. After dissolving formazan crystals with DMSO, the absorbance was measured at 570 nm using a microplate reader (Spectra Max M5; Molecular Devices; Sunnyvale, CA, USA). The cell variabilities of each group were determined by comparing them to those of the control cells, which were treated with 0.1% DMSO only.

### 2.4. Terminal Deoxynucleotidyl Transferase dUTP End Labeling (TUNEL) Staining Using a Cell Death Detection Kit

Apoptotic cell death was assessed via terminal deoxynucleotidyl transferase dUTP end labeling (TUNEL) staining using the Roche Cell Death Detection Kit (Roche Diagnostics, Manheim, BW, Germany) according to the manufacturer’s instructions. Briefly, the cells were pretreated with SA (100, 200, and 400 μM) for 24 h, and then treated with 50 μM 6-OHDA for another 12 h. Cells were then fixed with 4% paraformaldehyde for 30 min at room temperature. The cells were then observed using a fluorescence microscope (IX-81; Olympus Corp., Tokyo, Japan). The percentage of apoptotic cells was calculated as the ratio of TUNEL-positive nuclei to the total number of nuclei.

### 2.5. Hoechst 33,342 Staining

Nuclear staining of cells pretreated with SA (100, 200, and 400 μM) for 24 h followed by treatment with 50 μM of 6-OHDA for another 12 h was performed using the Hoechst 33,342 dye (Thermo Fisher Scientific Inc., Waltham, MA, USA) to detect apoptotic cells. Briefly, the cells were fixed with 4% paraformaldehyde for 30 min at room temperature and incubated with 10 μg/mL of Hoechst 33,342 dye for 30 min at 37 °C. The cells were then observed using a fluorescence microscope (IX-81; Olympus Corp.). The percentage of apoptotic cells was calculated as the ratio of condensed nuclei versus the total number of nuclei.

### 2.6. Measurement of Intracellular ROS Production

The analyses of ROS production was performed by using 2′,7′-dichlorodihydrofluorescin-diacetate (DCFH-DA; Thermo Fisher Scientific Inc., Waltham, MA, USA) dye according to the manufacturer’s instructions. Briefly, 1 × 10^5^ SH-SY5Y cells in 6-well plates were treated with 1 μM of DCF-DA dye for 30 min at 37 °C, after which they were washed twice with phosphate-buffered saline (PBS). The cells were observed using a fluorescence microscope equipped with iXon Electron Multiplying Charge-Coupled Device (EMCCD) cameras (Oxford Instruments, Oxfordshire, UK). Fluorescence was measured using a fluorescence spectrophotometer (Spectra Max M5) at 488 nm excitation and 515 nm emission wavelengths, respectively.

### 2.7. Measurement of Mitochondrial Membrane Potential (MMP)

MMP was measured via staining with the JC-1 assay kit (Thermo Fisher Scientific Inc.) according to the manufacturer’s instructions. Briefly, the cells were pretreated with SA (100, 200, and 400 μM) for 24 h followed by treatment with 50 μM of 6-OHDA for another 12 h, after which they were incubated with 10 μg/mL of JC-1 dye for 20 min at 37 °C. JC-1-stained cells were observed using a fluorescence microscope equipped with iXon EMCCD cameras (Oxford Instruments, Oxfordshire, UK). Green fluorescence intensity was measured using a fluorescence spectrophotometer (Spectra Max M5) at 550 nm excitation and 600 nm emission wavelengths, respectively, whereas red fluorescence was measured at 485 nm excitation and 535 nm emission wavelengths, respectively.

### 2.8. Western Blot Analysis

After pretreatment with SA (100, 200, and 400 μM) followed by treatment with 50 μM of 6-OHDA, the cells were harvested and lysed in RIPA buffer (LPS Solution, Daejeon, Korea) containing 150 mM of NaCl, 1% Triton X-100, 1% sodium deoxycholate, 0.1% SDS, 50 mM of Tris-HCl pH 7.5, and 2 mM of EDTA with a protease inhibitor cocktail (Thermo Fisher Scientific Inc.) and a phosphatase inhibitor cocktail (Roche Diagnostics). Protein homogenates were separated on 10% to 12% SDS-PAGE gels and then transferred to Polyvinylidene Difluoride (PVDF) membranes. After blocking with 5% bovine serum albumin (BSA) in 0.01 mol/L Tris-buffed saline (TBS) with 0.1% Tween 20 for 1 h at room temperature, the membranes were then incubated with primary antibodies overnight at 4 °C. Finally, the membranes were incubated with horseradish peroxidase-conjugated secondary antibodies (Jackson ImmunoResearch Lab., Inc., WestGrove, PA, USA) for 1 h at room temperature. The protein bands were visualized using an Immobilon Western Chemiluminescence kit (Millipore Corp., Billerica, MA, USA) and a UVITEC Mini HD9 system equipped with HD imaging software (Cleaver Scientific Ltd., Warwickshire, UK). Each protein band was then quantified using HD imaging software. The following were the primary antibodies used in this study: B-cell lymphoma 2 (Bcl-2; sc-492; 1:1000; Santa Cruz Biotechnology, Dallas, TX, USA); Bcl-2-associated X protein (Bax; #2772; 1:1000, cell signaling, Beverly, CA, USA); pro-caspase 3 (#9665; 1:1000, Cell Signaling); cleaved caspase 3 (#9664; 1:1000, cell signaling); superoxide dismutase1 (SOD1; sc-101523; 1:1000; Santa Cruz); SOD2 (#1341; 1:1000, cell signaling); catalase (#14097; 1:1000, Cell signaling); mitochondrial complex II protein (MS204; 1:1000, Abcam, Cambridge, UK); pancreatic ER kinase (PERK; #3192; 1:1000, cell signaling); phosphorylated PERK (p-PERK; ab192591; 1:1000, Abcam); eukaryotic translation initiation factor 2α (eIF2α; #9722; 1:1000, cell signaling); p-eIF2α (#3597; 1:1000, cell signaling); activating transcription factor 4 (ATF4; #11815; 1:1000, cell signaling); C/EBP homologous protein (CHOP; #2895; 1:1000, cell signaling); growth arrest and DNA damage-inducible protein45α (GADD45α; #4632; 1:1000, Cell signaling); KDEL (ab12223; 1:1000, Abcam) for detecting glucose regulated protein (GRP) 94 and 78 proteins; extracellular signal-regulated kinase 1/2 (ERK1/2; #9102; 1:1000; Cell Signaling), p-ERK1/2 (#9101; 1:1000; Cell Signaling), c-Jun N-terminal kinase (JNK; #9252; 1:1000; Cell Signaling), p-JNK (#3251; 1:1000; Cell Signaling), p-p38 (#9211; Cell Signaling), p38 (#9212; Cell Signaling), tyrosine hydroxylase (#2792; Cell Signaling), and β-actin (sc-47778; 1:1000; Santa Cruz).

### 2.9. Quantitative Real-Time Polymerase Chain Reaction (qRT-PCR)

Total RNA was extracted from cells that were pretreated with SA (100, 200, and 400 μM) followed by treatment with 50 μM of 6-OHDA using the Ribospin™ II kit (GeneAll Biotechnology Co., LTD, Seoul, Korea). After measuring the total RNA concentration using a UV/Vis Nano Spectrophotometer (MicroDigital Co., Ltd., Sungnam, Korea), 1 μg of total RNA from each group was reverse transcribed to synthesize cDNA using the GoScript Reverse Transcription System Kit (Promega Co., Madison, WI, USA). qRT-PCR was performed using a TaKara Thermal Cycler Dice Real-Time System (Takara Bio. Inc., Shiga, Japan) using the TOPreal™ qPCR 2× PreMIX Kit (Enzynomics Co., Daejeon, Korea). The primer sequences are as shown in Table 1.

### 2.10. Statistical Analyses

Statistical analyses were conducted via one-way analysis of variance (ANOVA) coupled with Bonferroni’s post-hoc test for multiple comparisons using GraphPad Prism 5.03 (GraphPad Software Inc., San Diego, CA, USA). For the normality test, the data were analyzed by column statistics with three normality tests, including Kolmogorov–Smirnov, D’Agostino and Pearson omnibus, and the Shapiro–Wilk normality test. If the data did not pass any normality test, Dunns post-hoc test with the Kruskal–Wallis test were further performed. All data were reported as the mean ± standard error of the mean (SEM). P-values < 0.05 were considered significant.

## 3. Results

### 3.1. Cytotoxic Effects of Sinapic Acid on SH-SY5Y Human Neuroblastoma Cells

**Figure 1:**
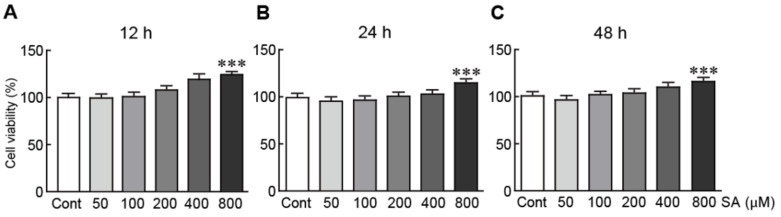
Cytotoxic effects of SA on SH-SY5Y neuroblastoma cells. Cell viability was measured via the MTT assay. The cells were pretreated with 50, 100, 200, 400, and 800 μM SA for 12 (**A**), 24 (**B**), and 48 h (**C**). The columns and error bars represent the mean ± standard error of the mean (SEM) from three independent experiments. Significance was determined via one-way ANOVA coupled with Bonferroni’s post hoc test. *** *p* < 0.001 vs. control group. Cont, control; SA, sinapic acid.

To determine the cytotoxicity of SA on SH-SY5Y cells, the viability of cells treated with various concentrations of SA (50, 100, 200, 400, and 800 μM) for 12, 24, and 48 h was assessed via the MTT assay. Cell viability remained stable in the presence of 50, 100, 200, 400, and 800 μM of SA, at any duration time (Figure 1). Interestingly, the 800 μM of SA treatment significantly increased cell viability after 12, 24, and 48 h, indicating that SA induces cell proliferation (42.5%, 15.9%, and 15.0% increases in cells pretreated with 800 μM of SA for 12 h, 24 h, and 48 h, respectively, relative to the control cells; Figure 1). Therefore, SA concentrations of 100, 200, and 400 μM were selected for downstream studies to elucidate the protective effects of SA on 6-OHDA-induced neurotoxicity in SH-SY5Y neuroblastoma cells.

### 3.2. Sinapic Acid Rescues SH-SY5Y Neuroblastoma Cells from 6-OHDA-Induced Neurotoxicity

**Figure 2:**
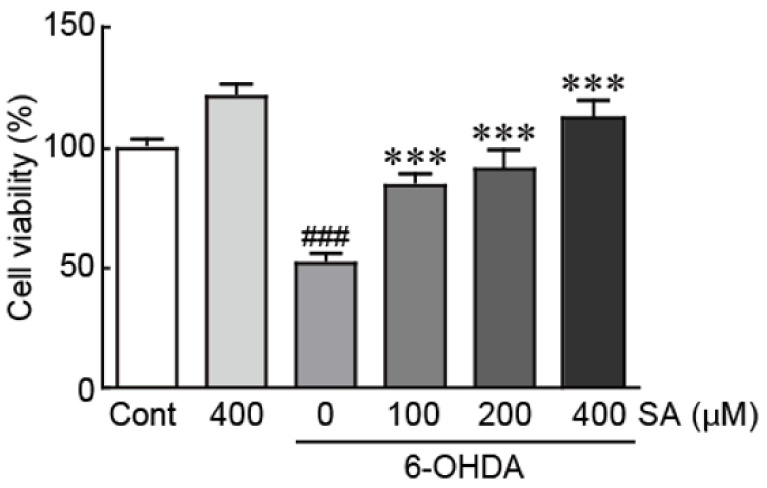
SA rescues SH-SY5Y neuroblastoma cells from 6-OHDA-induced neurotoxicity. The cell viability was measured via the MTT assay using cells treated with 6-OHDA for 24 h with or without 100, 200, or 400 μM of SA pretreatment for 24 h. The columns and error bars represent the mean ± standard error of the mean (SEM) from three independent experiments. Significance was determined via a one-way ANOVA coupled with Bonferroni’s post hoc test. ^###^ *p* < 0.001 vs. control group. *** *p* < 0.001 vs. 6-OHDA-only group. Cont, control; SA, sinapic acid; 6-OHDA, 6-hydroxydopamine.

To elucidate the protective effect of SA against 6-OHDA-induced neurotoxicity in SH-SY5Y cells, the viability of cells treated with 50 μM of 6-OHDA alone or pretreated with 100, 200, and 400 μM of SA for 24 h followed by 50 μM of 6-OHDA for another 24 h was assessed via the MTT assay. The viability of the cells treated with 50 μM of 6-OHDA alone was significantly reduced to 52.0% relative to the DMSO-treated (control) cells (Figure 2). In contrast, the viability of SA-pretreated cells was dramatically increased in a dose-dependent manner, reaching 84.6%, 91.5%, and 113.3% in cells pretreated with 100, 200, and 400 μM of SA, respectively, relative to the control cells (Figure 2). Therefore, our findings indicate that SA rescues SH-SY5Y neuroblastoma cells from cell death caused by 6-OHDA neurotoxicity.

In addition, cells were treated with 50 μM of rotenone, as another PD causing agent. Similarly, the viability of the cells treated with 50 μM of rotenone alone was significantly decreased to 57.2% compared with control cells. However, SA pretreatment was significantly preserved the viability of the cells (23.0%, 26.6%, and 38.6% increases in cells pretreated with 100, 200, and 400 μM of SA, respectively, relative to the rotenone alone-treated cells; Figure S1).

Furthermore, Western blot analysis was performed to test whether SA could preserve the expression of tyrosine hydroxylase (TH) protein, which is the rate-limiting enzyme that convers tyrosine to L-dopa, the precursor of dopamine, in SA-pretreated cells. The expression levels of TH protein were significantly decreased by 6-OHDA (0.4-fold decrease in 6-OHDA-treated cells relative to the controls). In contrast, the expression level of TH protein was preserved by SA pretreatment in a dose-dependent manner (0.3-, 0.4-, and 0.5-fold increases in the 100, 200, and 400 μM pretreated-cells compared to the 6-OHDA alone-treated cells, respectively; Figure S2).

### 3.3. Sinapic Acid Attenuates 6-OHDA-Induced Apoptotic Cell Death in SH-SY5Y Neuroblastoma Cells

**Figure 3:**
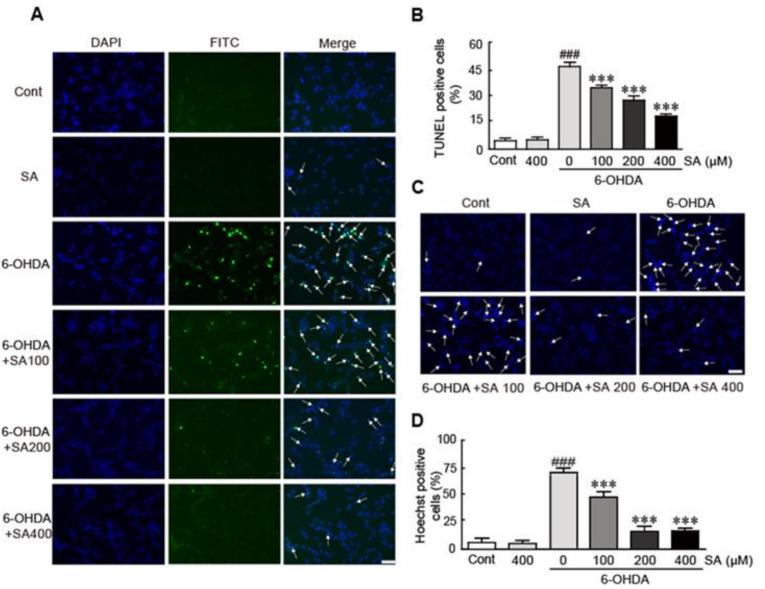

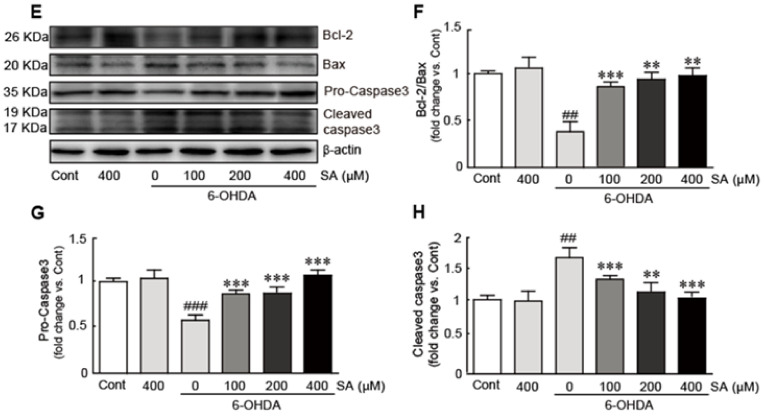
SA attenuates 6-OHDA-induced SH-SY5Y neuroblastoma cell apoptosis. Representative photograph of 6-OHDA-treated cells for 24 h with or without 100, 200, and 400 μM SA pretreatment for 24 h in the TUNEL (**A**) and Hoechst staining (**C**) assays. The apoptotic index was calculated by determining the percentage of TUNEL-positive (**B**) or Hoechst-positive (**D**) cells versus the total cells. The white arrow indicates TUNEL or apoptotic-positive cells. At least 30 cells per field were counted and 10 fields were determined in each group for TUNEL and Hoechst staining analysis. The cells were observed under 200× magnification. (**E**) Western blot analysis was performed to measure the expression levels of Bcl-2, Bax, pro-, and cleaved caspase 3 proteins in SA-pretreated/6-OHDA-treated cells. The expression levels of Bcl-2 (**F**), Bax (**F**), pro- (**G**), and cleaved (**H**) caspase 3 proteins were quantified with HD imaging software. β-actin was used as the loading control. Western blot analysis was performed in triplicate with three independent samples. All data were reported as the mean ± standard error of the mean (SEM). Significance was determined via a one-way ANOVA coupled with Bonferroni’s post hoc test. ^##^ *p* < 0.01 and ^###^ *p* < 0.001 vs. control group. ** *p* < 0.01 and *** *p* < 0.001 vs. 6-OHDA-only-treated group. Cont, control; SA, sinapic acid; 6-OHDA, 6-hydroxydopamine; Bcl-2, B-cell lymphoma 2; Bax, Bcl2-associated X protein. Scale bar, 100 μm.

To elucidate whether SA prevents apoptotic cell death in 6-OHDA-induced neurotoxicity, SH-SY5Y cells were treated with 100, 200, and 400 μM of SA for 24 h, after which they were treated with 50 μM of 6-OHDA for another 24 h. First, TUNEL staining was performed to detect the cells that were in the process of apoptosis. Our results demonstrated that 6-OHDA treatment significantly increased apoptotic cells, as demonstrated by a 47.3% apoptotic cell proportion (Figure 3A,B). However, the proportion of apoptotic cells was lower among all SA-pretreated cells (37.8%, 24.2%, and 15.3% in the 100, 200, and 400 μM SA-pretreated cells, respectively). Next, Hoechst 33,342 nuclear staining assay was performed to observe nuclear condensation, as this is among the main features of apoptotic cell death. Similar to the TUNEL staining assay results, the occurrence of cells with condensed nuclei was dramatically reduced by SA pretreatment, whereas 6-OHDA treatment significantly increased the proportion of cells with condensed nuclei (62.8% in the 6-OHDA only treatment versus 40.7%, 7.3%, and 4.3% in cells with 100, 200, and 400 μM SA-pretreatment, respectively) (Figure 3C,D). Finally, Western blot analysis was performed to determine the expression levels of apoptosis-related proteins, including Bcl-2, Bax, and caspase 3. The expression levels of the Bcl-2 protein (i.e., an anti-apoptotic protein) were significantly decreased by 6-OHDA (2.0-fold decrease in 6-OHDA-treated cells relative to the controls). In contrast, the expression level of Bax (i.e., a pro-apoptotic protein) was significantly increased in the 6-OHDA-only treated cells (0.3-fold decrease in Bcl-2/Bax in 6-OHDA-treated cells compared with that in control cells) (Figure 3E,F). Notably, SA pretreatment attenuated the effect of 6-OHDA-treatment on protein expression in a dose-dependent manner (0.8-, 0.9-, and 1.1-fold increases in the 100, 200, and 400 μM pretreated and 6-OHDA treated cells compared to the 6-OHDA alone-treated cells, respectively; Figure 3E,F). Additionally, the activation of caspase 3, a pro-apoptotic protein, was significantly inhibited by SA pretreatment, whereas caspase 3 was activated in the 6-OHDA-treated cells, resulting in decreases in pro-caspase 3 (i.e., the active form of this protein) and increases in cleaved caspase 3 (i.e., the inactive form of this protein), as demonstrated by pro- and cleaved caspase 3 Western blot analysis (0.6-fold decrease and 1.6-fold increase in pro- and cleaved caspase 3 in the 6-OHDA only cells compared to the control, respectively) (Figure 3E,G,H). However, SA pretreatment significantly attenuated these changes in pro- and cleaved caspase 3 protein expression (0.3- and 0.3-fold, and 0.7-fold increases in pro-caspase 3 and 0.3-, 0.4-, and 0.5-fold decreases in cleaved caspase 3 in the 100, 200, and 400 μM SA-pretreated cells compared with the 6-OHDA alone-treated cells, respectively) (Figure 3E,G,H). Collectively, our findings indicate that SA effectively prevents 6-OHDA-induced SH-SY5Y neuroblastoma cell apoptosis.

### 3.4. Sinapic Acid Attenuates Oxidative Stress Caused by 6-OHDA-Induced Neurotoxicity in SH-SY5Y Neuroblastoma Cells

**Figure 4:**
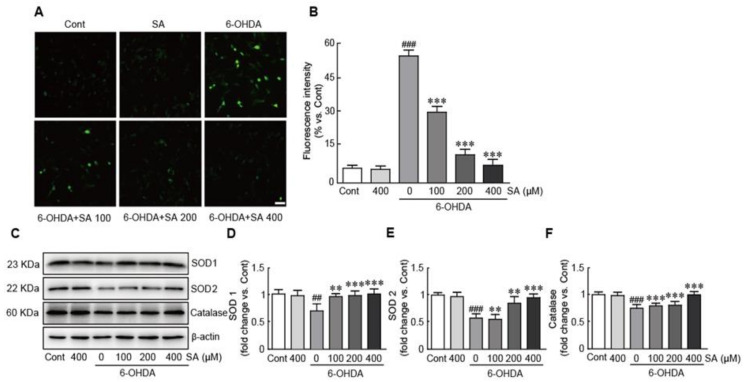
SA attenuates oxidative stress caused by 6-OHDA-induced neurotoxicity in SH-SY5Y neuroblastoma cells. Representative photograph of DCFH-DA staining assay (**A**) and fluorescence intensity (**B**) in cells treated with 6-OHDA for 24 h with or without 100, 200, and 400 μM SA pretreatment for 24 h. (**C**) Western blot analysis was performed to measure superoxide dismutase1 (SOD 1), SOD 2, and catalase protein expression in SA-pretreated/6-OHDA-treated cells. The expression levels of the SOD 1 (**D**), SOD 2 (**E**), and catalase (**F**) proteins were quantified using HD imaging software. β-actin was used as the loading control. Western blot analysis was performed in triplicate with three independent samples. All data are reported as the mean ± standard error of the mean (SEM). Significance was determined via a one-way ANOVA coupled with Bonferroni’s post hoc test. ^##^ *p* < 0.01 and ^###^ *p* < 0.001 vs. control group. ** *p* < 0.01 and *** *p* < 0.001 vs. 6-OHDA-only group. Cont, control; SA, sinapic acid; 6-OHDA, 6-hydroxydopamine; SOD, superoxide dismutase. Scale bar, 100 μm.

To determine whether SA attenuates oxidative stress, SH-SY5Y cells were pretreated with 100, 200, and 400 μM of SA followed by 50 μM 6-OHDA treatment. The cells were then incubated with DCFH-DA to measure ROS levels. Our findings indicated that 6-OHDA alone dramatically increased ROS production (57.7% increase compared with control cells). In contrast, when the cells were pretreated with SA, ROS production was significantly decreased in a dose-dependent manner (40.0%, 66.7%, and 80.0% decreases in 100, 200, 400 μM SA-pretreated cells compared with 6-OHDA alone-treated cells, respectively; Figure 4A,B). Furthermore, we determined the expression levels of antioxidant proteins, including SOD1, SOD2, and catalase. As expected, these proteins were significantly downregulated in the 6-OHDA-only-treated cells (0.6-, 0.4-, and 0.6-fold decreases in SOD1, SOD2, and catalase compared with the controls, respectively). However, the expression of these antioxidant proteins was rescued by SA pretreatment in a dose-dependent manner (Figure 4C–F). Therefore, our results demonstrate that SA exhibits a protective role against oxidative stress via the inhibition of ROS production and preservation of antioxidant protein expression in 6-OHDA-treated SH-SY5Y neuroblastoma cells.

### 3.5. Sinapic Acid Suppresses Mitochondrial Dysfunction Caused by 6-OHDA-Induced Neurotoxicity in SH-SY5Y Neuroblastoma Cells

**Figure 5:**
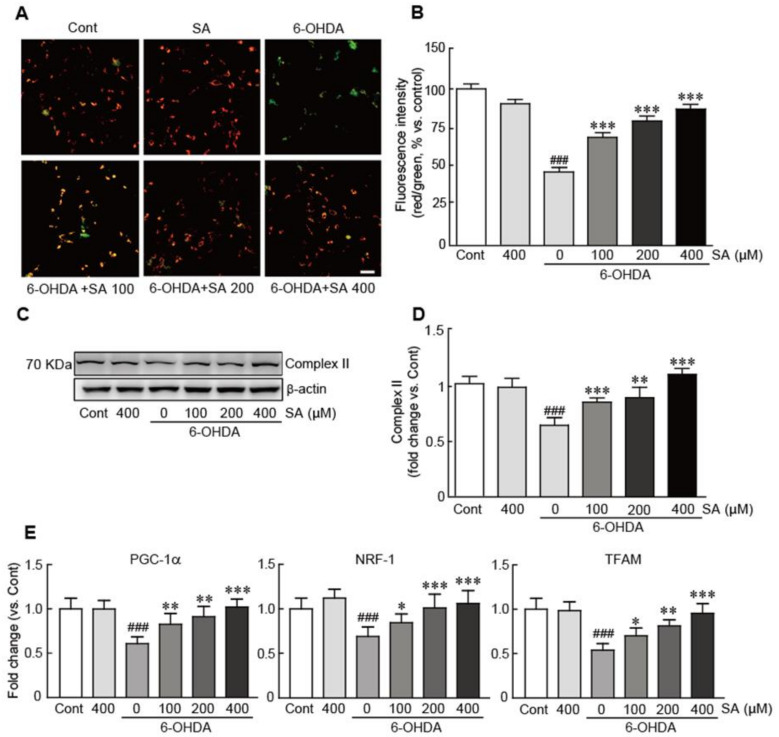
SA suppresses mitochondrial dysfunction caused by 6-OHDA-induced neurotoxicity in SH-SY5Y neuroblastoma cells. Representative photograph of the JC-1 staining assay (**A**) and red and green fluorescence intensities (**B**) in cells treated with 6-OHDA for 24 h with or without 100, 200, and 400 μM SA pretreatment for 24 h. (**C**) Western blot analyses were performed to measure the expression of mitochondrial complex II protein in SA-pretreated/6-OHDA-treated cells. (**D**) The expression level of mitochondrial complex II protein was quantified using HD imaging software. β-actin was used as the loading control. (**E**) qRT-PCR analysis was performed to determine the mRNA expression levels of the mitochondrial biogenesis genes PGC-1α, NRF-1, and TFAM. Western blot and qRT-PCR analysis were performed in triplicate with three independent samples. All data are reported as the mean ± standard error of the mean (SEM). Significance was determined via a one-way ANOVA coupled with Bonferroni’s post hoc test. ^###^ *p* < 0.001 vs. control group. * *p* < 0.05, ** *p* < 0.01, and *** *p* < 0.001 vs. 6-OHDA-only-treated group. Cont, control; SA, sinapic acid; 6-OHDA, 6-hydroxydopamine; Complex II, mitochondrial complex II protein; PGC-1α, peroxisome proliferator-activated receptor-γ coactivator-1α; NRF-1, nuclear respiratory factor-1; TFAM, mitochondrial transcription factor A. Scale bar, 100 μm.

To determine the preventive effect of SA on mitochondrial dysfunction caused by 6-ODHA treatment, the JC-1 staining assay was performed to measure MMP (i.e., an indicator of mitochondrial function) in cells treated with 6-OHDA with or without 100, 200, and 400 μM SA pretreatment. MMP collapsed in the 6-OHDA-only-treated cells, as demonstrated by their prominent green fluorescence, whereas control cells largely exhibited red fluorescence (i.e., 40% decrease in the percentage of red/green fluorescence intensity compared with the control cells). Conversely, when pretreated with SA, red fluorescence was gradually replenished in a dose-dependent manner, indicating that SA effectively preserved MMP (21.8%, 30.9%, and 45.5% increases in 100, 200, and 400 μM SA-pretreated cells compared with the6-OHDA alone-treated cells, respectively; Figure 5A,B). Additionally, the expression of mitochondrial complex II protein was also preserved by SA pretreatment, whereas 6-OHDA significantly decreased the expression level of this protein (0.6-fold decrease in 6-OHDA-alone-treated cells compared to the control cells and 0.2-, 0.3-, and 0.6-fold increases in 100, 200, and 400 μM SA-pretreated cells compared to the 6-OHDA alone-treated cells, respectively; Figure 5C,D). Finally, qRT-PCR was performed to elucidate the effect of SA on the expression of several genes involved in mitochondrial biogenesis, including peroxisome proliferator-activated receptor-γ coactivator-1α (PGC-1α), nuclear respiratory factor-1 (NRF-1), and mitochondrial transcription factor A (TFAM). The mRNA levels of these genes were significantly decreased when the cells were treated with 6-OHDA (0.6-, 0.7-, and 0.5-fold decreases in PGC-1α, NRF-1, and TFAM in 6-OHDA-only-treated cells compared with the control cells, respectively). However, SA treatment replenished the mRNA expression levels of all these genes in a dose-dependent manner (Figure 5E). Collectively, these results indicate that SA efficiently attenuates the mitochondrial dysfunction caused by 6-OHDA-induced neurotoxicity in SH-SY5Y neuroblastoma cells.

### 3.6. Sinapic Acid Orevents ER Stress Caused by 6-OHDA-Induced Neurotoxicity in SH-SY5Y Neuroblastoma Cells

**Figure 6:**
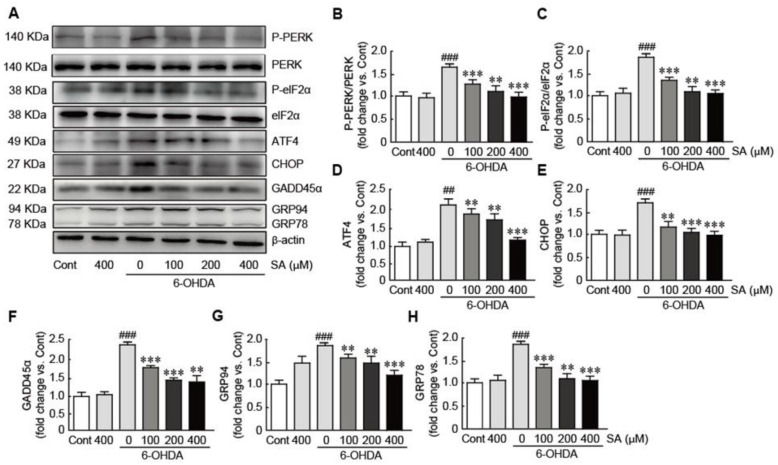
SA prevents ER stress caused by 6-OHDA-induced neurotoxicity in SH-SY5Y neuroblastoma cells. (**A**) Western blot analyses were performed to measure the expression levels of ER-stress-related proteins in SA-pretreated/6-OHDA-treated cells. The expression levels of total and phospho-PERK (**B**), total and phospho-eIF2α (**C**), ATF4 (**D**), CHOP (**E**), GADD45α (**F**), GRP94 (**G**), and GRP78 (**H**) proteins were quantified using HD imaging software. β-actin was used as the loading control. Western blot analysis was performed in triplicate with three independent samples. Data are shown as the mean ± standard error of the mean (SEM). Significance was determined via a one-way ANOVA with a Bonferroni post hoc test. ^##^ *p* < 0.01 and ^###^ *p* < 0.001 vs. control group. ** *p* < 0.01 and *** *p* < 0.001 vs. the 6-OHDA-only-treated group. Cont, control; SA, sinapic acid; 6-OHDA, 6-hydroxydopamine; PERK, pancreatic ER kinase; eIF2α, eukaryotic translation initiation factor 2α; ATF4, activating transcription factor 4; CHOP, C/EBP homologous protein; GADD45α, growth arrest and DNA damage-inducible protein45α; GRP94 and 78, glucose regulated protein94 and 78.

To evaluate whether SA prevents 6-OHDA-induced ER stress responses, Western blot analysis was performed against various proteins involved in the ER stress signaling pathway, including PERK, eIF-2α, ATF, CHOP, GADD45α, and GRP94 and GRP78. In 6-OHDA-only-treated cells, phosphorylated PERK and EIF2α was significantly upregulated, indicating that PERK and eIF2a were activated (1.6- and 1.9-fold increases in p-PERK and p-eIF2α in 6-OHDA-only-treated cells compared with control cells, respectively; Figure 6A–C). Additionally, the expression levels of the ATF, CHOP, GADD45α, and GRP94 and GRP78 proteins were also significantly upregulated in 6-OHDA-only-treated cells (2.1-, 1.7-, 2.2-, 1.8-, and 1.7-fold increases in ATF, CHOP, GADD45α, and GRP94 and GRP78 in 6-OHDA-only-treated cells compared with those in control cells, respectively; Figure 6A,D–H). Notably, SA pretreatment dramatically downregulated the expression levels of the aforementioned proteins in a dose-dependent manner. Notably, the expression levels of these proteins in the 200 and 400 μM SA-treated cells were similar to those in the control cells. Therefore, our results indicate that SA effectively inhibits the ER-stress induced by 6-OHDA in SH-SY5Y neuroblastoma cells.

### 3.7. Sinapic Acid Inhibits the Activation of the MAPK Signaling Pathway Caused by 6-OHDA-Induced Neurotoxicity in SH-SY5Y Neuroblastoma Cells

**Figure 7:**
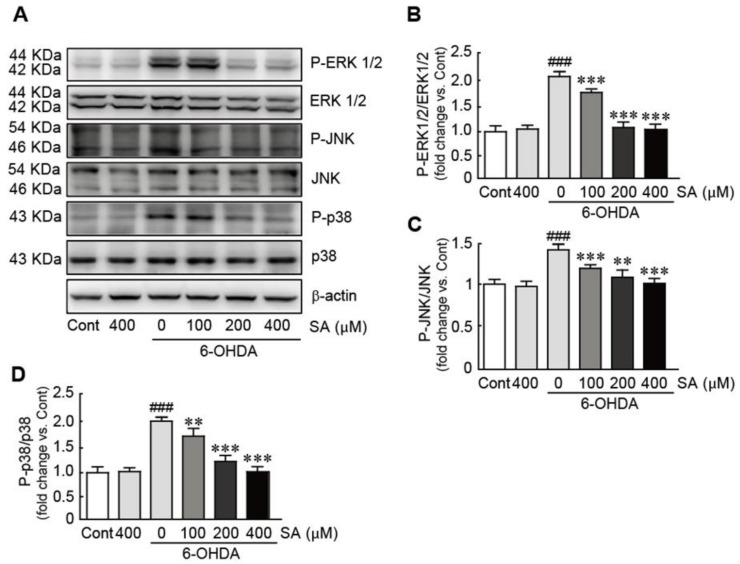
SA inhibits the activation of the MAPK signaling pathway caused by 6-OHDA-induced neurotoxicity in SH-SY5Y neuroblastoma cells. (**A**) Western blot analysis was performed to measure the expression levels of MAPK proteins in SA-pretreated/6-OHDA-treated cells. The expression levels of total and phospho-ERK 1/2 (**B**), total and phospho-JNK (**C**), and total and phospho-p38 (**D**) proteins were quantified using HD imaging software. β-actin was used as the loading control. Western blot analyses were performed in triplicate with three independent samples. All data were reported as the mean ± standard error of the mean (SEM). Significance was determined via one-way ANOVA coupled with Bonferroni’s post hoc test. ^###^ *p* < 0.001 vs. control group. ** *p* < 0.01 and *** *p* < 0.001 vs. the 6-OHDA-only-treated group. Cont, control; SA, sinapic acid; 6-OHDA, 6-hydroxydopamine.

To investigate the effect of SA on the mitogen-activated protein kinase (MAPK) signaling pathway during 6-OHDA-induced neurotoxicity, Western blot analysis was performed against three typical proteins involved in the MAPK signaling pathway, including ERK1/2, JNK, and p38. Our results demonstrated that ERK1/2, JNK, and p38 protein phosphorylation was significantly increased in 6-OHDA-only-treated cells (2.1-, 1.4-, and 2.1-fold increases in p-ERK1/2, p-JNK, and p-p38 compared with the control cells; Figure 7). In contrast, these proteins were dramatically downregulated in a dose-dependent manner when the cells were pretreated with SA (Figure 7). Therefore, these results indicate that SA effectively attenuated the activation of the MAPK signaling pathway in 6-OHDA-treated SH-SY5Y neuroblastoma cells.

## 4. Discussion

6-OHDA is a hydroxylated dopamine analog that enters the cells via the dopamine transporter and accumulates intracellularly thereafter [29]. In turn, this compound causes serious cellular damages, including mitochondrial dysfunction, oxidative stress, and apoptosis, thereby inducing the selective degeneration of catecholaminergic neurons, particularly dopaminergic neurons [30]. Due to its neurotoxic effects, 6-OHDA has been commonly used to create PD disease models for both in vitro and in vivo studies since it was first identified in 1959 [31,32]. Therefore, our study used 6-OHDA to induce neurotoxicity in SH-SY5Y human neuroblastoma cells to explore the protective effects of SA against PD.

We first sought to evaluate cell viability to determine whether SA rescues the cells from 6-OHDA-induced neurotoxicity. Our findings demonstrated that 6-OHDA decreases cell viability, whereas SA pre-treatment dramatically replenished cell viability in a dose-dependent manner. We then examined the protective effects of SA against 6-OHDA-induced apoptosis. Our TUNEL and Hoechst 33,342 assays demonstrated that SA pretreatment dramatically reduced the occurrence of apoptotic cells, which exhibit DNA fragmentation and nuclear condensation. Additionally, SA pretreatment also significantly reduced the expression of pro-apoptotic proteins such as Bax and caspase 3, and increased Bcl-2 protein expression (i.e., an anti-apoptotic protein) in 6-OHDA-treated cells. These results suggest that SA protects SH-SY5Y neuronal cells from 6-OHDA-induced neurotoxicity.

Over the past three decades, mitochondrial dysfunction has been believed to be a crucial initiating factor responsible for PD pathogenesis [33]. Indeed, most PD cases are sporadic and stem from environmental and genetic susceptibility [34]. For instance, exposure to neurotoxins such as MPTP and rotenone, a commonly used pesticide, has been linked to dopaminergic neuron damage and Parkinsonian symptoms. Both of these compounds are typical mitochondrial complex I inhibitors, and therefore inhibit critical cellular functions associated with energy production [35,36]. Additionally, PD genetic studies have identified several genes associated with monogenic forms of PD, such as α-synuclein, Parkin, PTEN-induced putative kinase 1 (PINK1), and DJ-1, all of which are closely related to mitochondrial dysfunction [37,38]. Therefore, our findings indicate that preserving mitochondrial function is an effective means to prevent and treat PD. Based on these observations, we then sought to determine whether SA could protect mitochondrial functions against 6-OHDA-induced neurotoxicity. Our results demonstrated that SA pretreatment preserved MMP and mitochondrial complex II protein expression, both of which are involved in the electron transport chain process for energy production, whereas 6-OHDA led to MMP collapse and the downregulation of mitochondrial complex II protein expression. Furthermore, the expression of mitochondrial metabolic genes, including PGC-1-α, NRF-1, and TFAM, were also rescued in 6-OHDA-treated cells. These findings prove that SA prevents 6-OHDA-induced mitochondrial dysfunction in SH-SY5Y cells.

Mitochondrial dysfunction induces the generation of reactive oxygen species (ROS), which leads to oxidative damage (i.e., oxidative stress) within the cells. Many studies have demonstrated that oxidative stress plays a crucial role in PD pathogenesis. Therefore, many mitochondrial-specific antioxidant compounds such as mitoquinone [39], mitoapocynin [40], and melatonin [41] have been identified and their PD preventive effects have been elucidated. Particularly, SA is a well-known naturally occurring antioxidant, and many studies have demonstrated its preventive effects against many diseases [42]. Our previous study also demonstrated that SA exerts protective effects against cardiac hypertrophy in phenylephrine-induced rat cardiomyocytes via the inhibition of mitochondrial damage and oxidative stress [27]. Similarly, the present study revealed that SA efficiently inhibits ROS overproduction and increases the expression of several endogenous antioxidants such as SOD 1, SOD 2, and catalase against 6-OHDA-induced neurotoxicity.

Evidence from clinical studies suggests that the pathogenesis of PD is closely linked to the activation of ER stress. In post-mortem brain tissues from PD patients, accumulation of ER chaperones and increases in PERK and eIF2a protein expression was identified in Lewy bodies, which are abnormal protein deposits in the dopaminergic neurons of the substantia nigra [43,44]. Notably, mutated forms of the Parkin and leucin-rich repeat kinase 2 (LRRK2) genes, which are involved in the inhibition of ER stress, have been associated with familial PD cases. Parkin is upregulated and provides cytoprotective effects after treatment with ER stressors in neuronal cells [45]. Additionally, overexpression of Parkin was also found to protect cells against ER stress [46]. Interestingly, LRRK2 abrogation has been reported to protect neuronal cells both in vitro and in vivo [47]. Therefore, the suppression of ER stress is important to preserve the function of neuronal cells against PD progression. Here, we sought to determine the preventive effect of SA on ER stress by examining the expression of several proteins involved in the ER stress signaling pathway such as PERK, eIF2α, ATF4, CHOP, and GADD45α. We found that the PERK and eIF2α proteins were activated by 6-OHDA treatment, whereas this effect was significantly reversed via SA pretreatment. Furthermore, the ATF, CHOP, and GADD45α proteins, which are downstream of PERK and eIF2α, were also upregulated in 6-ODHA-treated cells. Notably, the expression levels of these proteins were also dramatically attenuated in SA-pretreated cells in the presence of 6-OHDA. Therefore, our results indicate that SA rescues the cells by effectively blocking ER stress caused by 6-OHDA-induced neurotoxicity.

MAPK regulates many physiological and pathological processes such as gene expression, metabolism, cell differentiation, stress-related responses, and apoptotic cell death [48]. Some studies have also indicated that the MAPK signaling pathway is activated by a variety of pathological stimuli such as ROS, ER stress, and lipopolysaccharide [49]. Moreover, α-synuclein and LRRK2, both of which are known to contribute to PD occurrence, are associated with the activation of the MAPK signaling pathway [50,51]. Therefore, we hypothesized that the PD preventive effects of SA are mediated via the MAPK signaling pathway, possibly by inhibiting the activation of this signaling pathway. Our findings demonstrate that SA could significantly inhibit the activation of typical MAPK proteins, such as ERK1/2, JNK, and p38, whereas these MAPK proteins were activated in 6-OHDA-treated cells.

In conclusion, the present study demonstrated that SA attenuates 6-OHDA-induced neurotoxicity in SH-SY5Y neuroblastoma cells (a PD in vitro model). Furthermore, our study determined that the neuroprotective effects of SA were mediated by the suppression of oxidative stress and ER stress. Therefore, our findings highlight the potential of SA as a therapeutic agent for the prevention and treatment of PD. Furthermore, the preventive effect of SA against PD should be further determined using differentiated SH-SY5Y cells induced by retinoic acid and PD-induced animal models to overcome limitation of presented results using un-differentiated SH-SY5Y neuroblastoma cells.

## Figures and Tables

**Table 1:** Quantitative real-time polymerase chain reaction (qRT-PCR) primer sequences.

Genes	Primers	
PGC-1α	forward	5′-TCA GTC CTC ACT GGT GGA CA-3′
	reverse	5′-TGC TTC GTC GTC AAA AAC AG-3′
NRF-1	forward	5′-CTA CTC GTG TGG GAC AGC AA-3′
	reverse	5′-AAT TCC GTC GAT GGT GAG AG-3′
TFAM	forward	5′-GGC ACA GGA AAC CAG TTA GG-3′
	reverse	5′-CAG AAC ACC GTG GCT TCT AC-3′
18S rRNA	forward	5′-GAG CGA AAG CAT TTG CCA AG-3′
	reverse	5′-GGC ATC GTT TAT GGT CGG AA-3′

PGC-1α, peroxisome proliferator-activated receptor-γ coactivator-1α; NRF-1, nuclear respiratory factor-1; TFAM, mitochondrial transcription factor A.

## Supplementary Materials

The following are available online at https://www.mdpi.com/2227-9059/9/3/295/s1, Figure S1: SA rescues SH-SY5Y neuroblastoma cells from rotenone-induced neurotoxicity, Figure S2: SA preserves the expression level of tyrosine hydroxylase protein caused by 6-OHDA-induced neurotoxicity in SH-SY5Y neuroblastoma cells.
