# Supplementary material for: Sinapic Acid Protects SH-SY5Y Human Neuroblastoma Cells against 6-Hydroxydopamine-Induced Neurotoxicity
Source: Biomedicines. 2021 Mar 13;9(3):295. doi: 10.3390/biomedicines9030295 (PMC8000777; doi:10.3390/biomedicines9030295)
Supplement: Supplementary file 1 [file biomedicines-09-00295-s001.pdf]

biomedicines

MDPI

Supplementary Materials

Sinapic Acid Protects SH-sy5y Human Neuroblastoma Cells against 6-Hydroxydopamine-Induced Neurotoxicity

Tsendsuren Tungalag and Dong Kwon Yang *

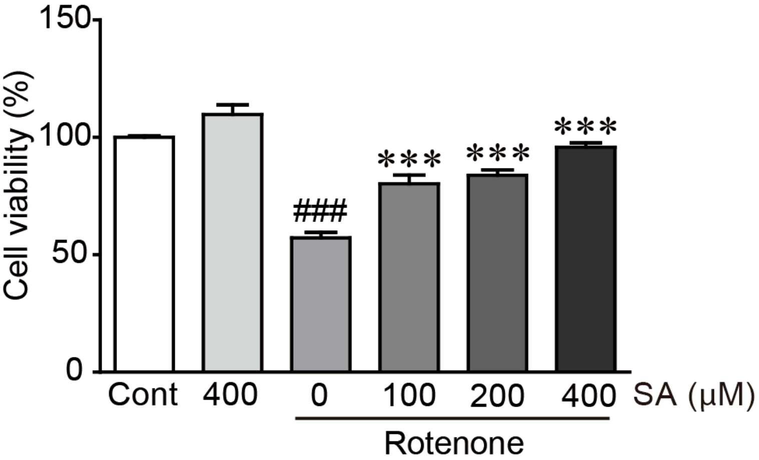

Figure S1. SA rescues SH-SY5Y neuroblastoma cells from rotenone-induced neurotoxicity. The cell viability was measured via the MTT assay using cells treated with rotenone for 24 h with or without 100, 200, or 400 μM SA pretreatment for 24 h. The columns and error bars represent the mean ± standard error of the mean (SEM) from three independent experiments. Significance was determined via a one-way ANOVA coupled with Bonferroni's post hoc test. ###P < 0.001 vs. control group. ***P < 0.001 vs. rotenone-only group. Cont, control; SA, sinapic acid.

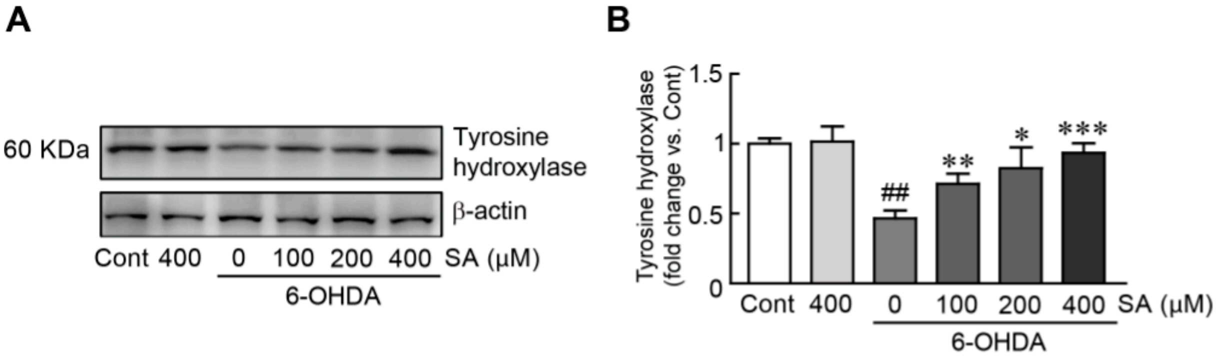

Figure S2. SA preserves the expression level of tyrosine hydroxylase protein caused by 6-OHDA-induced neurotoxicity in SH-SY5Y neuroblastoma cells. (**A**) Western blot analyses were performed to measure the expression level of tyrosine hydroxylase protein in SA-pretreated/6-OHDA-treated cells. (**B**) The expression level of tyrosine hydroxylase protein was quantified using HD imaging software. β-actin was used as the loading control. Western blot analysis was performed in triplicate with three independent samples. Data are shown as the mean ± standard error of the mean (SEM). Significance was determined via a one-way ANOVA with a Bonferroni post hoc test. ##P < 0.01 vs. control group. *P < 0.05, **P < 0.01, and ***P < 0.001 vs. the 6-OHDA-only-treated group. Cont, control; SA, sinapic acid; 6-OHDA, 6-hydroxydopamine.
